# Supplementary material for: Structure-Based Functional Analysis of a Hormone Belonging to an Ecdysozoan Peptide Superfamily: Revelation of a Common Molecular Architecture and Residues Possibly for Receptor Interaction
Source: Int J Mol Sci. 2021 Oct 15;22(20):11142. doi: 10.3390/ijms222011142 (PMC8541221; doi:10.3390/ijms222011142)
Supplement: Supplementary file 1 [file ijms-22-11142-s001.zip › ijms-1378068-supplementary.pdf]

International Journal of
Molecular Sciences

MDPI

Supporting Information

Structure-Based Functional Analysis of a Hormone Belonging to an Ecdysozoan Peptide Superfamily: Revelation of a Common Molecular Architecture and Residues Possibly for Receptor Interaction

Yun-Ru Chen [1], Nai-Wan Hsiao [2], Yi-Zong Lee [1,3], Shiau-Shan Huang [2], Chih-Chun Chang [2], Jyuan-Ru Tsai [4], Hui-Chen Lin [4], Jean-Yves Toullec [5], Chi-Ying Lee [2,*] and Ping-Chiang Lyu [1,6,*]

1 Institute of Bioinformatics and Structural Biology, National Tsing Hua University, Hsinchu 300044, Taiwan; chenyrusa@gmail.com (Y.-R.C.); s942545@go.thu.edu.tw (Y.-Z.L.)
2 Department of Biology, National Changhua University of Education, Changhua 500207, Taiwan; nady@cc.ncue.edu.tw (N.-W.H.); hss.33333@gmail.com (S.-S.H.); u9120037@imss.hshs.tyc.edu.tw (C.-C.C.)
3 Instrument Center, National Tsing Hua University, Hsinchu 300044, Taiwan
4 Department of Life Science, Tunghai University, Taichung 407224, Taiwan; jrtsaibio@thu.edu.tw (J.-R.T.); hclin@thu.edu.tw (H.-C.L.)
5 Faculté de Sciences, CNRS, UMR 7144, Adaptation et Diversité en Milieu Marin, AD2M, Station Biologique de Roscoff, Sorbonne Université, 29682 Roscoff, France; jean-yves.toullec@sb-roscoff.fr
6 Department of Medical Sciences, National Tsing Hua University, Hsinchu 300044, Taiwan
* Correspondence: bicylee@cc.ncue.edu.tw (C.-Y.L.); pclyu@mx.nthu.edu.tw (P.-C.L.); Tel.: +886-4724-7756 (C.-Y.L.); +886-3574-2762 (P.-C.L.); Fax: +886-4721-1156 (C.-Y.L.); +886-3571-5934 (P.-C.L.)

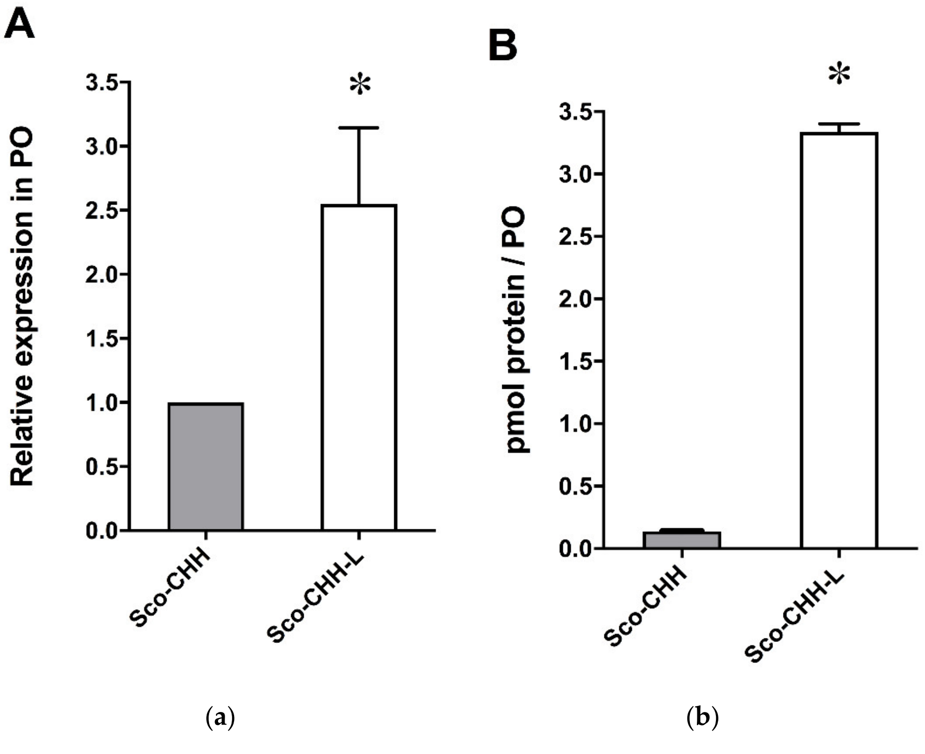

Figure S1. Preferential expression of Sco-CHH-L in the pericardial organs of the mud crab Scylla olivacea**.** (**A**) Levels of Sco-CHH and Sco-CHH-L transcript determined by a semi-quantitative real-time PCR. Data are means ± SEM. n = 4 for each group. Transcript levels are normalized to a reference gene (18s rRNA) and expressed relative to the Sco-CHH levels. (**B**) Quantification of Sco-CHH and Sco-CHH-L by an ELISA using purified Sco-CHH-L and Sco-CHH as standards. The asterisk (*) indicates significant difference at 5% level.

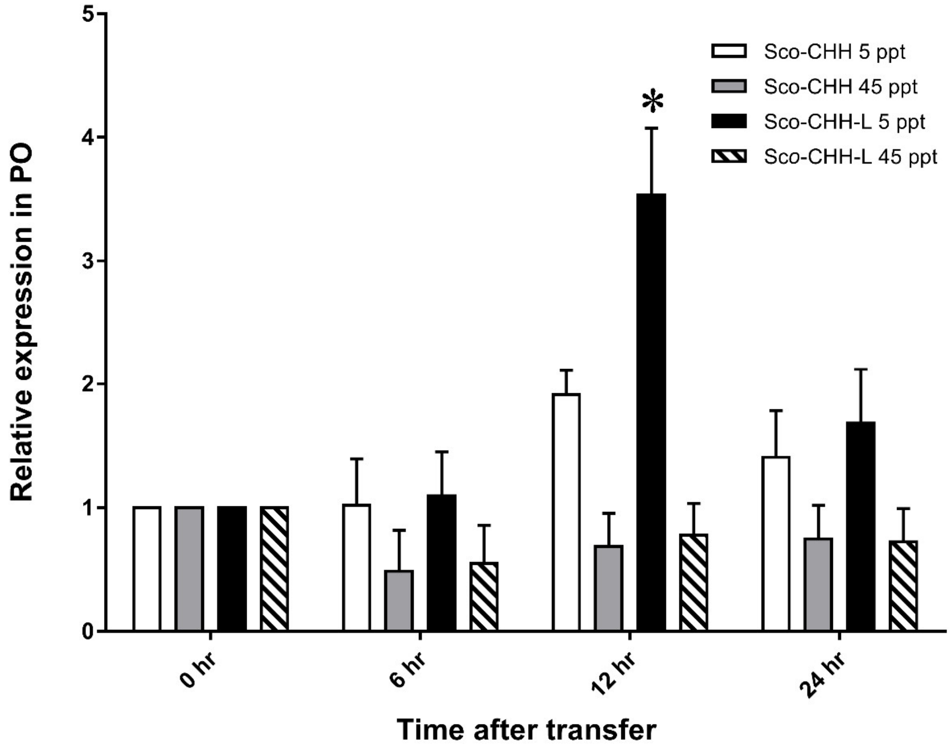

Figure S2. Pattern of changes in Sco-CHH and Sco-CHH-L transcript levels in the pericardial organ of the mud crab Scylla olivacea in response to osmotic stresses. Pericardial organs were harvested from 25 ppt-acclimated animals before (0 h) or at the designated time points (6, 12, 24 h) after being transfer to a hypo-osmotic (5 ppt) or hyper-osmotic (45 ppt) environment and processed for total RNA extraction and reverse transcription reaction. Transcripts levels were estimated by a semi-quantitative real-time PCR. Data are means ± SEM. *n* = 4 for each time point. Transcript levels are normalized to a reference gene (18s rRNA) and expressed relative to the respective control (0 h) levels. The asterisk (*) indicates significantly different from respective 0-h control at 5% level.

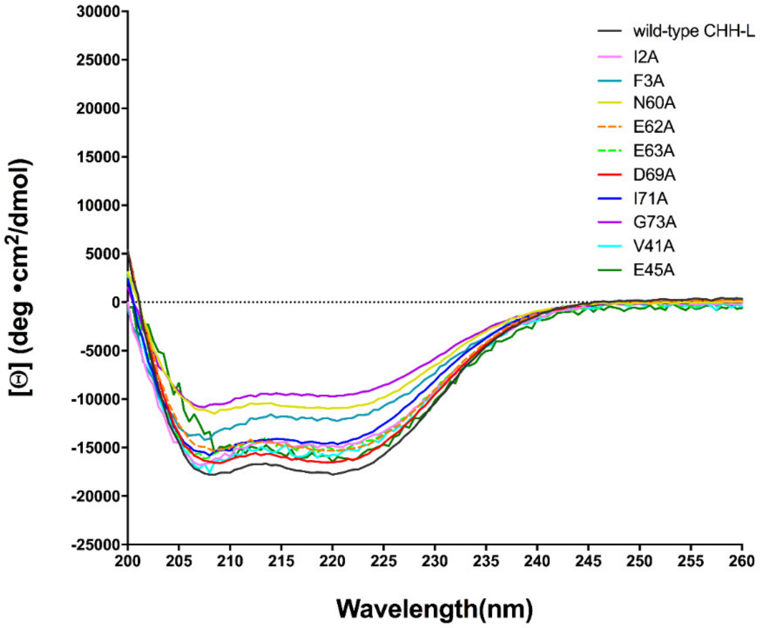

Figure S3. Far-UV circular dichroism spectra of the wild-type rSco-CHH-L and ala-nine-substituted mutants. CD spectra of the purified peptides (each 15 μM) in phosphate buff-ered saline were recorded at 25 °C with a wavelength range of 260 to 200 nm, using a 1-mm path length quartz cell.

Table S1. NMR restraints and structural statistics for crustacean hyperglycemic hormone-like peptide (Sco-CHH-L) from Scylla olivacea in solution.

**Experimental constraints**	
NOE	1123
Intra-residue	338
Sequential (｜I-J｜ =1)	337
Medium range (｜I-J｜ ≦ 4)	371
Long range (｜I-J｜) ＞ 4)	77
Hydrogen bond constraints	50
Dihedral angle constraints	94
Φ	47
Ψ	47
**RMSD (Å) with respect to the average structure**	
Well-defined regions (res. 9-28, 34-38, 43-54, 62-72)	
Backbone	0.54 ± 0.14
Heavy atoms	1.43 ± 0.27
All residues (res. 1-74)	
Backbone	1.42 ± 0.51
Heavy atoms	2.31 ± 0.51
**RMSD from idealized covalent geometry**	
Bonds (Å)	0.0174 ± 0.0005
Angles (°)	2.1032 ± 0.0496
Impropers (°)	2.5548 ± 0.0148
**Ramachandran statistics**	
Most favored regions	75.4%
Additionally allowed regions	23.2%
Generously allowed regions	1.4%
Disallowed regions	0.0%

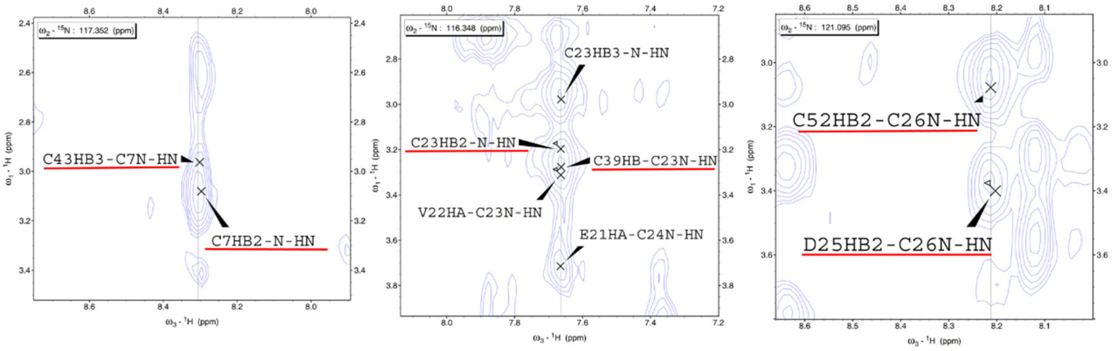

Figure S4. NMR data confirming disulfide bridge connectivity. The three disulfide bonds (Cys[7]–Cys[43], Cys[23]–Cys[39] and Cys[26]–Cys[52]; left to right) of Sco-CHH-L were confirmed by the corresponding HSQC-NOESY spectrum cross-peaks between Hβ protons of cysteine residues.

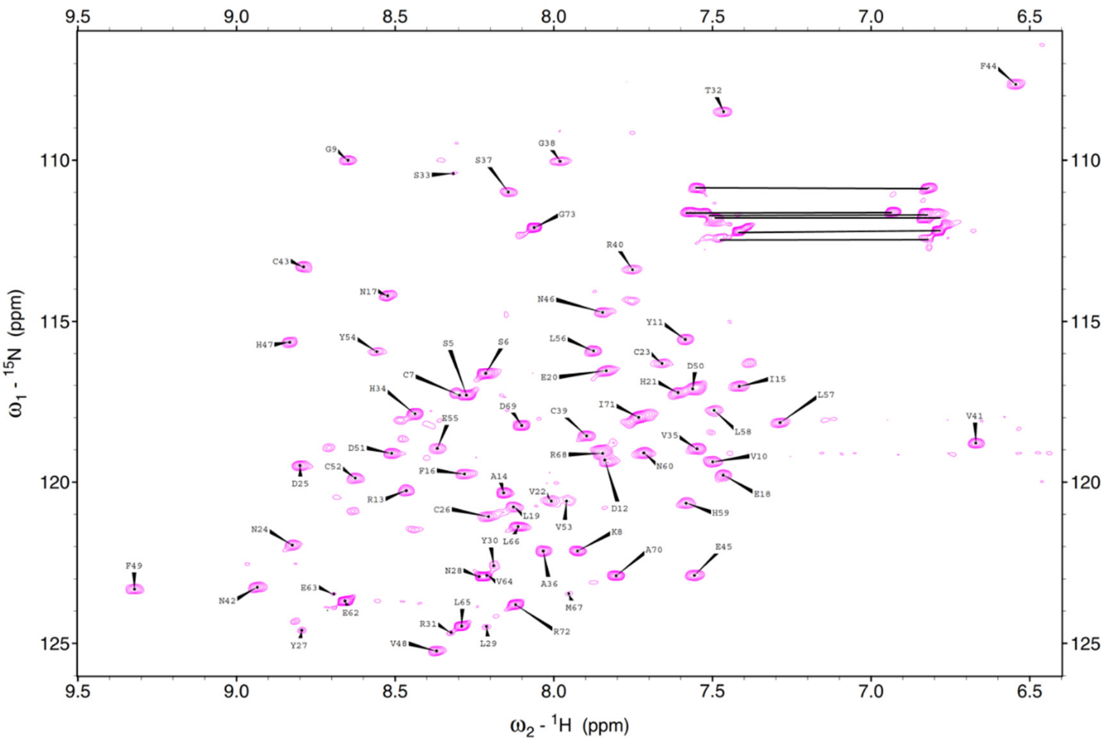

Figure S5. Assignment of amide resonances of Sco-CHH-L on a ^{1}H-^{15}N HSQC spectrum. Horizontal lines connect pairs of peaks corresponding to sidechain amide groups of Asn and Gln residues. All backbone amide peaks are well resolved, except for Q1, I2, F3, D4 and P62.

Table S2. Synthetic oligonucleotides used in this study.

	**A. Real-time PCR Primers**
**Name**	Sequence (from 5′ to 3′)
18s-F	TGGTGCATGGCCGTTCTTA
18s-R	AATTGCTGGAGATCCGTCGAC
Sco-CHH_F	GTGGGTGCAGATCTAACTGCTACAG
Sco-CHH_R	GCGCCTATGGTGAATCTTTA
Sco-CHH-L_F	CAGAACTTCCCATGTCGCTAGTGGGT
Sco-CHH-L_R	CCTCCTCCGGATTGTGCAGCAG
	**B. Sco-CHH-L Mutagenesis Primes**
Name	Sequence (from 5′ to 3′)
I2A_F	GATATACATATGCAGGCGTTCGATTCTTCGTG
I2A_R	CACGAAGAATCGAACGCCTGCATATGTATATC
F3A_F	CATATGCAGATCGCGGATTCTTCGTG
F3A_R	CACGAAGAATCCGCGATCTGCATATG
V41A_F	GCTAGTGGGTGCAGAGCGAACTGTTTCGAAAAC
V41A_R	GTTTTCGAAACAGTTCGCTCTGCACCCACTAGC
E45A_F	GCAGAGTGAACTGTTTCGCGAACCATGTATTTGATGAC
E45A_R	GTCATCAAATACATGGTTCGCGAAACAGTTCACTCTGC
N60A_F	GAACTACTGCTGCACGCGCCGGAGGAGGTACTG
N60A_R	CAGTACCTCCTCCGGCGCGTGCAGCAGTAGTTC
E62A_F	CTGCTGCACAATCCGGCGGAGGTACTGCTTATG
E62A_R	CATAAGCAGTACCTCCGCCGGATTGTGCAGCAG
E63A_F	CACAATCCGGAGGCGGTACTGCTTATG
E63A_R	CATAAGCAGTACCGCCTCCGGATTGTG
D69A_F	GTACTGCTTATGAGAGCGGCCATCAGGG
D69A_R	CCCTGATGGCCGCTCTCATAAGCAGTAC
I71A_F	CTTATGAGAGACGCCGCGAGGGGTTAACTCGAG
I71A_R	CTCGAGTTAACCCCTCGCGGCGTCTCTCATAAG
G73A_F	CGCCATCAGGGCGTAACTCGAGCAC
G73A_R	GTGCTCGAGTTACGCCCTGATGGCG
